# Analgesic Efficacy of Bromelain and Bromelain Plus Turmeric for Pain Control After Orthodontic Separator Placement: A Triple‐Blind Randomized Clinical Trial

**DOI:** 10.1002/cre2.70124

**Published:** 2025-04-22

**Authors:** Shabnam Ajami, Seyyed Hadi Hosseini, Neda Babanouri, Zahra Hashemi

**Affiliations:** ^1^ Orthodontic Research Center, School of Dentistry Shiraz University of Medical Sciences Shiraz Iran; ^2^ Postgraduate student Orthodontic Research Center, School of Dentistry Shiraz University of Medical Sciences Shiraz Iran; ^3^ School of Dentistry Shiraz University of Medical Sciences Shiraz Iran

**Keywords:** Bromelains, curcumin, Ibuprofen, orthodontics, corrective, pain management

## Abstract

**Objectives:**

This study aimed to compare the analgesic efficacy of Bromelain, Ibuprofen, and Bromelain plus turmeric for pain control after orthodontic separator placement.

**Material and Methods:**

This triple‐blind randomized clinical trial included 135 patients over the age of 15 who required orthodontic treatment. The patients were randomly assigned to three groups: 400 mg Ibuprofen (Group A), 200 mg Bromelain (ANAHEAL; Group B), and 150 mg Bromelain + 300 mg Turmeric (ANAHEAL PLUS; Group C). The patients took one tablet immediately after the separator was placed. Four separators were placed at the mesial and distal regions of both maxillary first molars. The degree of pain and discomfort was measured using a visual analog scale (VAS) at the following time points: Immediately after the separator placement (T_0_), 2 h posttreatment (T_1_), 6 h posttreatment (T_2_), 24 h posttreatment (T_3_), and 48 h after separator placement (T_4_). The data were analyzed using SPSS (version 15.0). Different statistical tests including ANOVA, Tukey's test, and paired *t*‐test were utilized. *p* < 0.05 was considered statistically significant.

**Results:**

Pain scores at 2 h (T1) and 6 h (T2) after separator placement differed significantly among the three groups (*p* = 0.006 and *p* = 0.025, respectively), with the Ibuprofen group experiencing significantly higher pain levels than the Bromelain and Bromelain Plus Turmeric groups. At 48 h (T4), a significant difference was observed only in chewing function (*p* = 0.024), where patients in the Bromelain group reported higher pain scores than those in the Ibuprofen group. Sex had no significant effect on pain perception (*P* < *0.05*).

**Conclusions:**

Bromelain with or without turmeric might be a useful alternative to Ibuprofen for pain control following orthodontic separator placement.

## 1 Materials and Methods

### 1.1 Trial Design

A single‐center, triple‐blind randomized clinical trial was conducted at the Department of Orthodontics, School of Dentistry, Shiraz University of Medical Sciences (Shiraz, Iran) for 6 months from 2020 to 10‐06. The study was approved by the Ethics Committee of Shiraz University of Medical Sciences, (IR.SUMS.DENTAL.REC.1399.067). The trial was registered at the Iranian Registry of Clinical Trials (IRCT20181121041713N3). The present study was a three‐arm parallel trial with 1:1:1 allocation. The applied method remained unchanged throughout the study.

### 1.2 Participants, Eligibility Criteria, and Settings

The target population comprised patients of both genders referred to the orthodontic clinic at the School of Dentistry for fixed orthodontic treatment. The inclusion criteria consisted of (i) the necessity for orthodontic separator placement to commence orthodontic treatment of the maxillary arch, (ii) an age exceeding 15 years, (iii) the absence of current usage of antibiotics, analgesics, anti‐inflammatory medications, anticoagulants, diuretics, oral antidiabetics, lithium, cyclosporine, or methotrexate, (iv) no requirement for antibiotic prophylaxis, and (v) no pregnancy or lactation.

The exclusion criteria were (i) extraction or missing of any maxillary tooth except third molars, (ii) smoking, (iii) chronic systemic diseases or coagulation disorders, (iv) contraindications for NSAIDs, Bromelain, or turmeric, and (v) allergy to pineapple. Before the study, the participants were informed about the research objectives, intervention procedures, and potential risks and benefits. Moreover, We prioritized voluntary involvement and assured the confidentiality of any supplied information. Every single participant gave their written informed consent.

### 1.3 Sample Size Calculation

The sample size was calculated to be 39 patients in each group, assuming the mean pain score after a single dose prescription of Ibuprofen to be 3.19 ± 1.1, alpha = 0.05, beta = 0.2, and study power of 80%. To compensate for the potential dropouts, 45 patients were enrolled in each group.

### 1.4 Randomization Procedure

To allocate patients to three experimental groups with a 1:1:1 ratio, the block randomization method was implemented using the online software *RANDOM.ORG* and a nine‐block randomization scheme. Consequently, the random sequences of the study groups were concealed in opaque envelopes and shuffled before the intervention to enhance the unpredictability of the random allocation sequence. Each patient was requested to select a sealed envelope that would be assigned to one of the study groups. To mitigate selection bias, allocation concealment was implemented.

#### 1.4.1 Blinding

To ensure that all those involved in the study, including patients, researchers, and statisticians, were not aware of which group they were in, all of the pills in each group were covered with the same gelatin. Everyone else participating in the study—the statistician, the patients, and the clinicians—was in the dark about the group assignment; only the first author knew.

#### 1.4.2 Interventions

Separator placement for all patients was carried out by the same orthodontist (Alastiks S‐2separator modules lot number A2508, American Orthodontics, Washington, USA). Four separators were placed at the mesial and distal regions of both maxillary first molars. The patients were divided into three groups:

**Group A** (control): Patients received 400 mg Ibuprofen (ADVALGIN; Abidipharms, Iran).
**Group B**: Patients received 200 mg Bromelain (ANAHEAL 500; Salamat Parmoon Amin, Iran).
**Group C**: Patients received 150 mg Bromelain + 300 mg turmeric (ANAHEAL PLUS, Salamat Parmoon Amin, Iran).

All patients received only one tablet immediately after the separator placement.

##### 1.4.2.1 Primary and Secondary Outcomes

Patients' pain and discomfort levels were measured at several time points using the visual analog scale (VAS): Immediately after separator placement (T_0_), 2 h posttreatment (T_1_), 6 h posttreatment (T_2_), 24 h posttreatment (T_3_), and 48 h after separator placement (T_4_). A booklet including five VAS forms was given to each patient. The VAS was a 10‐cm line, where 0 denoted no pain and 10 the highest level of discomfort. On the VAS form, the patients received instructions to indicate their level of pain by drawing a short vertical line while they were at rest, chewing, and occluding the posterior. They were also told to submit the questionnaire at their subsequent appointment, which was set for a week later.

To occlude the posterior teeth, the patients were instructed to clench their teeth with a light force and refrain from eating anything throughout the procedure. Patients were asked to chew on a piece of green apple and record their pain levels on a visual analog scale (VAS) for the chewing function. The patients were instructed not to use any other analgesics during the study period. Those who used other analgesics were excluded from the study. In case of unbearable pain, the patients were asked to record the time and number of tablets consumed. Following the start of the experiment, the outcome measures remained unchanged.

### 1.5 Interim Analyses and Stopping Guidelines

No stopping guidelines were defined, and no interim assessments were conducted.

### 1.6 Statistical Analysis

The data were analyzed using SPSS version 15.0 (SPSS Inc., Chicago, IL, USA). ANOVA was applied to compare the pain scores between the groups. Repeated measures of ANOVA and paired *t*‐tests were used to analyze the differences in pain scores at different time points. Pairwise comparisons were performed using Tukey's post hoc test. *p* < 0.05 was considered statistically significant.

## 2 Results

### 2.1 Participant Flow

Fourteen patients were excluded from the study; out of which, 4 patients used other analgesics during the study, 5 patients did not complete the questionnaire correctly, and 5 did not return the questionnaire.

### 2.2 Baseline Data

**Figure 1:**
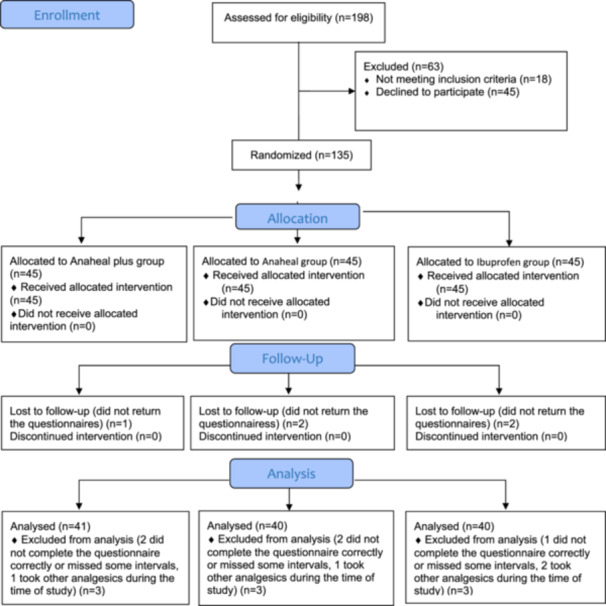
CONSORT flow diagram.

The final sample included 48 men (39.6%) and 73 women (60.4%), with 28 women and 13 men in the ANAHEAL PLUS group, 25 women and 15 men in the ANAHEAL group, and 20 women and 20 men in the Ibuprofen group. The three groups were not significantly different regarding sex distribution (*p* = 0.245). The mean age was 19.92 ± 4.82 years in the ANAHEAL PLUS group, 23.03 ± 6.65 years in the ANAHEAL group, and 21.97 ± 7.50 years in the Ibuprofen group. The three groups were not significantly different regarding the mean age (*p* = 0.999). Figure 1 shows the CONSORT flow diagram of the study.

### 2.3 Outcome

**Figure 2:**
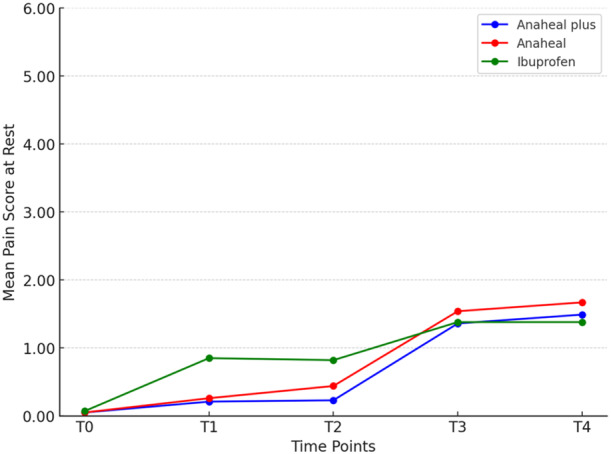
Comparison of the mean pain scores on VAS among the three study groups over the time in the rest position.

**Figure 3:**
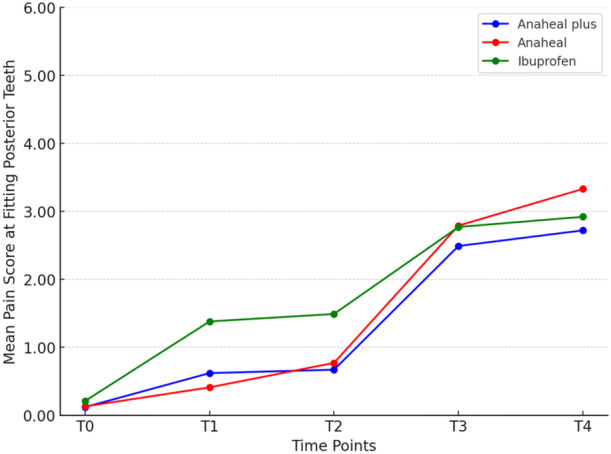
Comparison of the mean pain scores on VAS among the three study groups over the time when fitting posterior teeth.

**Figure 4:**
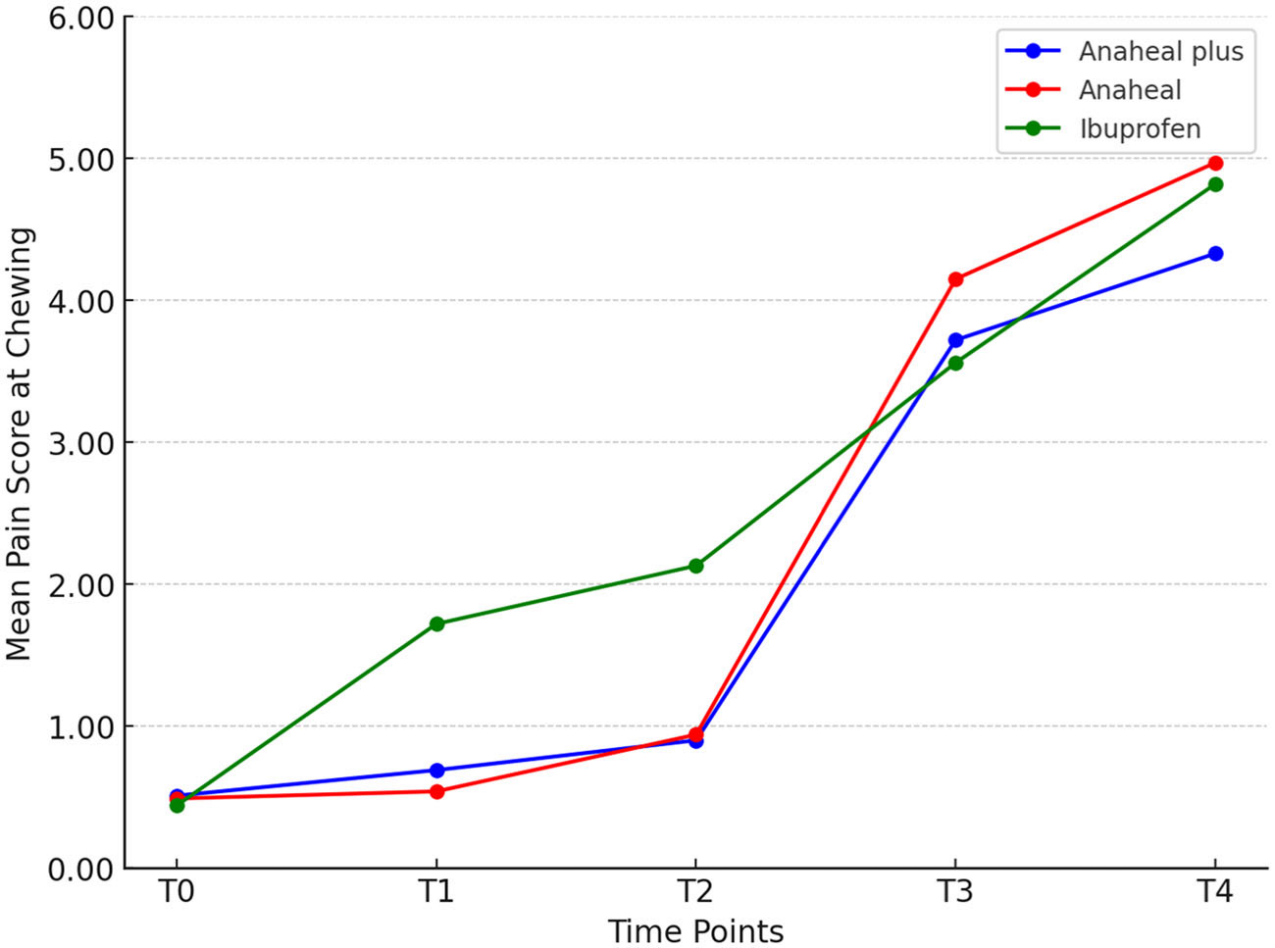
Comparison of the mean pain scores on VAS among the three study groups over the time in chewing function.

Table 1 presents the descriptive data and ANOVA results. Significant differences in pain scores were observed at T_1_ and T_2_ across all functions (*p* < 0.05), with the Ibuprofen group experiencing higher pain than both Bromelain‐based groups. At T_4_, the only significant difference was in chewing function (*p* = 0.024). The Tukey's test showed that at T_4_, the ANAHEAL group experienced greater pain than the Ibuprofen group (*p* = 0.017). No significant difference was observed between the ANAHEAL and ANAHEAL PLUS groups at T_1_ and T_2_ (Table 2). In general, chewing and occluding posterior teeth caused more pain than resting at all time points, with the lowest pain levels recorded at rest (Tables 3 and 4; Figures 2, 3, 4).

**Table 1:** Mean pain score of patients in the three groups in different functions at different time points.

Function	Group	T_0_, Mean ± SD	T_1_, Mean ± SD	T_2_, Mean ± SD	T_3_, Mean ± SD	T_4_, Mean ± SD
**Rest**	ANAHEAL PLUS	0.05 ± 0.12	0.21 ± 0.70	0.23 ± 0.49	1.36 ± 0.96	1.49 ± 0.89
	ANAHEAL	0.05 ± 0.22	0.26 ± 0.64	0.44 ± 0.85	1.54 ± 1.39	1.67 ± 1.42
	Ibuprofen	0.07 ± 0.35	0.85 ± 1.37	0.82 ± 1.34	1.38 ± 1.25	1.38 ± 1.55
	***p*‐value**	**0.893**	**0.006** *	**0.025** *	**0.78**	**0.633**
**Fitting Posterior**	ANAHEAL PLUS	0.12 ± 0.33	0.62 ± 0.96	0.67 ± 0.70	2.49 ± 1.43	2.72 ± 1.28
	ANAHEAL	0.13 ± 0.34	0.41 ± 0.79	0.77 ± 1.22	2.79 ± 2.01	3.33 ± 1.88
	Ibuprofen	0.21 ± 0.21	1.38 ± 1.70	1.49 ± 1.78	2.77 ± 1.74	2.92 ± 1.66
	***p*‐value**	**0.561**	**0.001** *	**0.013** *	**0.689**	**0.239**
**Chewing**	ANAHEAL PLUS	0.51 ± 0.50	0.69 ± 0.77	0.90 ± 0.82	3.72 ± 1.61	4.33 ± 1.56
	ANAHEAL	0.49 ± 0.51	0.54 ± 0.91	0.94 ± 1.32	4.15 ± 2.21	4.97 ± 1.93
	Ibuprofen	0.44 ± 0.50	1.72 ± 2.22	2.13 ± 2.17	3.56 ± 1.71	4.82 ± 1.99
	***p*‐value**	**0.791**	**0.001** *	**0.001** *	**0.352**	**0.024** *

*Note:* T_0_: immediately after separator placement; T_1_: 2 h after separator placement; T_2_: 6 h after separator placement; T_3_: 24 h after separator placement; T_4_: 48 h after separator placement. One‐way ANOVA test.

* Statistically significant.

**Table 2:** Pairwise comparisons of the groups regarding the mean pain score at T_1_ and T_2_.

Function	Group	T_1_	T_2_
**Rest**	ANAHEAL – ANAHEAL PLUS	0.970	0.612
	ANAHEAL PLUS – Ibuprofen	0.011*	0.020*
	ANAHEAL – Ibuprofen	0.021*	0.182
**Occluding posterior**	ANAHEAL – ANAHEAL PLUS	0.736	0.936
	ANAHEAL PLUS – Ibuprofen	0.016*	0.018*
	ANAHEAL – Ibuprofen	0.002*	0.045*
**Chewing**	ANAHEAL – ANAHEAL PLUS	0.887	0.988
	ANAHEAL PLUS – Ibuprofen	0.007*	0.002*
	ANAHEAL – Ibuprofen	0.001*	0.003*

*Note:* T_1_: 2 h after separator placement; T_2_: 6 h after separator placement. Tukey's post‐hoc test.

* Statistically significant.

**Table 3:** Mean pain scores in different functions at different time points.

Group	Function	T_0_, Mean ± SD	T_1_, Mean ± SD	T_2_, Mean ± SD	T_3_, Mean ± SD	T_4_, Mean ± SD
**ANAHEAL PLUS**	Rest	0.05 ± 0.12	0.21 ± 0.70	0.23 ± 0.49	1.36 ± 0.96	1.49 ± 0.89
	Occluding	0.12 ± 0.33	0.62 ± 0.96	0.67 ± 0.70	2.49 ± 1.43	2.72 ± 1.28
	Chewing	0.51 ± 0.50	0.69 ± 0.77	0.90 ± 0.82	3.72 ± 1.61	4.33 ± 1.56
	***p*‐value**	**< 0.001** *	**< 0.001** *	**< 0.001** *	**< 0.001** *	**< 0.001** *
**ANAHEAL**	Rest	0.05 ± 0.22	0.26 ± 0.64	0.44 ± 0.85	1.54 ± 1.39	1.67 ± 1.42
	Occluding	0.13 ± 0.34	0.41 ± 0.79	0.77 ± 1.22	2.79 ± 2.01	3.33 ± 1.88
	Chewing	0.49 ± 0.51	0.54 ± 0.91	0.94 ± 1.32	4.15 ± 2.21	4.97 ± 1.93
	***p*‐value**	**< 0.001** *	**0.036** *	**< 0.001** *	**< 0.001** *	**< 0.001** *
**Ibuprofen**	Rest	0.07 ± 0.35	0.85 ± 1.37	0.82 ± 1.34	1.38 ± 1.25	1.38 ± 1.55
	Occluding	0.21 ± 0.21	1.38 ± 1.70	1.49 ± 1.78	2.77 ± 1.74	2.92 ± 1.66
	Chewing	0.44 ± 0.50	1.72 ± 2.22	2.13 ± 2.17	3.56 ± 1.71	4.82 ± 1.99
	***p*‐value**	**0.01** *	**< 0.001** *	**< 0.001** *	**< 0.001** *	**< 0.001** *

*Note:* T_0_: immediately after separator placement; T_1_: 2 h after separator placement; T_2_: 6 h after separator placement; T_3_: 24 h after separator placement; T_4_: 48 h after separator placement. One‐way ANOVA test.

* Statistically significant.

**Table 4:** Comparison of the mean pain scores in different functions at different time points.

Group	Function	T_0_	T_1_	T_2_	T_3_	T_4_
**ANAHEAL PLUS**	Rest – occluding posterior teeth	0.787	< 0.001*	< 0.001*	< 0.001*	< 0.001*
	Rest – chewing	< 0.001*	0.002*	< 0.001*	< 0.001*	< 0.001*
	Occluding posterior teeth – chewing	< 0.001*	0.999	0.015*	< 0.001*	< 0.001*
**ANAHEAL**	Rest – occluding posterior teeth	0.787	0.971	0.001*	< 0.001*	< 0.001*
	Rest – chewing	< 0.001*	0.132	< 0.001*	< 0.001*	< 0.001*
	Occluding posterior teeth – chewing	< 0.001*	0.070*	0.19	< 0.001*	< 0.001*
**Ibuprofen**	Rest – occluding posterior teeth	0.505	0.12	0.003*	< 0.001*	< 0.001*
	Rest – chewing	0.001*	0.009*	< 0.001*	< 0.001*	< 0.001*
	Occluding posterior teeth – chewing	0.081	0.107	0.019*	0.017*	0.004*

*Note:* T_0_, immediately after separator placement; T_1_: 2 h after separator placement; T_2_: 6 h after separator placement; T_3_: 24 h after separator placement; T_4_: 48 h after separator placement. Tukey's post‐hoc test.

* Statistically significant.

Sex had no significant effect on pain perception across treatment groups or time periods except for occluding posterior teeth at T_3_ in the ANAHEAL group and T_4_ in the Ibuprofen group.

Pain perception scores differed significantly at different times (*p* = 0.001). However, For all three functions, the trend was nearly identical across all groups. The effect of each medicine on pain perception over time is detailed separately.

In the ANAHEAL group, the paired *t*‐test showed significant differences between T_1_–T_3_, T_2_–T_3_, T_1_–T_4_, and T_2_–T_4_ while occluding posterior teeth and chewing (*p* < 0.001).

In the ANAHEAL PLUS group, the pain increased from T_2_ to T_3_ (*p* < 0.001) at rest and while occluding posterior teeth and chewing. There was no significant difference from T_1_ to T_2_ or between T_3_ to T_4_ at rest and while occluding posterior teeth (*p* > 0.05). In chewing function, the pain score increased significantly from 2 h after separator placement and reached a peak at 48 h.

In the Ibuprofen group, the mean pain score increased significantly from 2 h after the separator placement and reached a peak at 48 h. The paired t‐test showed significant changes in pain score between T_1_–T_2_ (*p* = 0.046), T_1_–T_3_, T_1_–T_4_, T_2_–T_3_, T_2_–T_4_ (*p* < 0.001), and T_3_–T_4_ (*p* = 0.003) at rest. The pain score increased significantly from 6 h after separator placement to a peak at 24 h at rest. In chewing and occluding posterior teeth, there was no change in pain score from T_1_ to T_2_ (*p* = 0.999 and 0.147) and T_3_ to T_4_ (*p* = 0.999 and 0.908). The results of the paired *t*‐test showed significant changes between T_1_–T_3_, T_1_–T_4_, T_2_–T_3_, and T_2_–T_4_ (*p* < 0.001) (Figures 3 and 4).

## 3 Discussion

The present study assessed the analgesic efficacy of ANAHEAL tablets containing 200 mg Bromelain and ANAHEAL PLUS tablets containing 150 mg Bromelain + 300 mg Turmeric (95% curcumin) in comparison with 400 mg Ibuprofen for pain relief following the placement of orthodontic separators. A VAS was used in this study for the quantification of pain because it is a reliable and valid tool for the assessment of acute and chronic pain (Bird et al. 2007).

The findings revealed that, in general, the pain increased immediately after the placement of separators, reached its peak after 24 h in the majority of patients, and remained almost constant for 48 h. However, Najafi et al. (2015) and Abdaljawwad et al. (2016) reported that the level of pain reached its peak 24 h after the placement of separators, and decreased within 48 h. However, in the present study, the pain did not subside after 24 h. Nevertheless, the difference in the mean pain score at 24 and 48 h was not statistically significant.

Generally, patients in all groups and at all‐time points experienced higher levels of pain when chewing compared with rest and while occluding their posterior teeth. Similarly, Farzanegan et al. (2012), Young et al. (2006), and Thejasri et al. (2023) assessed the pain scores and reported that chewing caused the highest pain when compared with rest and occlusion of posterior teeth, which was similar to the results of the present study. This result was expected since orthodontic pain is caused by compression, inflammation, and edema in the PDL, and compression increases during function in the PDL (Farzanegan et al. 2012; Young et al. 2006).

Various studies have investigated both pharmacological and nonpharmacological methods for orthodontic pain management. According to the recent systematic review (Al‐Hanbali et al. 2024), low‐level laser therapy (LLLT) has been identified as one of the most effective nonpharmacological interventions, significantly reducing pain, particularly at 6 and 24 h after orthodontic procedures. Additionally, vibration devices and chewing interventions (such as chewing gum or bite wafers) have shown contradictory findings, with some studies reporting short‐term pain relief but limited long‐term benefits.

Several studies have shown that Ibuprofen is more effective than placebo in treating orthodontic pain relief. (Hosseinzadeh Nik et al. 2016; Polat et al. 2005; Sudhakar et al. 2014) for this reason, in the present study, we didn't have the placebo group and only comparison was conducted between Ibuprofen, ANAHEAL, and ANAHEAL PLUS. Cheng et al. (2020) showed that the maximum analgesic effect of Ibuprofen was after 6 h. Similarly, in our study, pain significantly increased between 6 and 24 h, which shows that the analgesic effect of the drug has been decreased.

In all positions, the mean pain score in the ANAHEAL and ANAHEAL PLUS groups was significantly lower than that in the Ibuprofen group at 2 and 6 h, indicating that Bromelain and curcumin were more effective than Ibuprofen in reducing pain after separator placement. However, there was no significant difference between the ANAHEAL and ANAHEAL PLUS groups, indicating that adding turmeric to Bromelain had no additional pain reduction benefit. Also, Bromelain has an added advantage and can be effective against SARS‐CoV‐2 virus that currently has become very important from a scientific, economic, and medical point of view (Hikisz and Bernasinska‐Slomczewska 2021). A recent meta‐analysis showed that Bromelain was significantly reducing pain scores than active control and placebo (Leelakanok et al. 2023). This result is in accordance with what has been reported in our study.

In a study by Viganò et al. Bromelain was found to be more effective than paracetamol and codeine in decreasing inflammation, edema, and trismus after oral surgical procedures (Viganò et al. 2017). Liu et al. conducted a meta‐analysis and evaluated the efficacy of Bromelain on facial pain and swelling in patients following third molar surgery, and reported that Bromelain was useful for the treatment of facial swelling in the early and late phases after surgery (Liu et al. 2019). Bromelain reduced pain 7 days post‐surgery by facilitating the return of interstitial fluid and inflammatory cells to the bloodstream, while simultaneously diminishing edema and lowering levels of prostaglandin E2 and substance P (Kyrkanides et al. 2000).

There are controversial reports concerning the efficacy of curcumin in alleviating pain in the orofacial region (Brignardello‐Petersen 2019; Meghana et al. 2020). Maulina et al. investigated the effect of curcuminoids on inflammation following third molar extraction and found that patients in the curcumin group experienced less pain than those in the Mefenamic acid group. They found that curcumin was beneficial in alleviating acute inflammatory pain following the surgical extraction of third molars (Maulina et al. 2018). The administration of antibiotics following third molar extraction may be responsible for alleviating postoperative pain and edema at the surgical sites. However, a current systematic review suggests that curcumin can be considered as an alternative to conventional analgesics for pain relief in orofacial region (Sterniczuk et al. 2022).

Previous studies have demonstrated that separator type significantly impacts pain perception and separation efficiency. Tripathi et al. (2019) and Kumar et al. (2022) found that Kesling separators provide adequate separation while causing the least pain, whereas Elastomeric separators, despite offering greater separation, resulted in significantly higher pain scores and discomfort. These findings align with the results of the present study, which confirmed that chewing function induces the highest pain levels, regardless of the separator type or analgesic intervention.

The findings of the present study also indicated that there was no difference in pain scores between men and women at rest or while chewing in the three groups. However, when the posterior teeth were occluded, the mean pain score in the ANAHEAL group was higher in women at 24 h, and the mean pain score was higher in men in the Ibuprofen group at 48 h. Except for the two aforementioned occasions, the pain scores were not significantly different between men and women. This conclusion is consistent several other studies that have examined orthodontic pain (Abdelrahman et al. 2015; Ertan Erdinc 2004; Jones and Chan 1992). Pain is a subjective response influenced by factors such as age, sex, level of pain threshold, the amount of force applied, cultural background, previous traumatic experiences, psychological factors, and the patient's mental state (Bergius et al. 2002; Bird et al. 2007; Kluemper et al. 2002). Thus, variations in the results of studies on this topic can be somehow expected.

All of the aforementioned studies documented the anti‐inflammatory and analgesic properties of Bromelain and curcumin. Besides, they recommended using them as alternatives to NSAIDs due to having fewer side effects. NSAIDs are contraindicated in several conditions such as gastrointestinal and renal disorders and may delay the rate of tooth movement. in all of these cases, Bromelain and curcumin can be used as alternatives for orthodontic pain management (Cooper 1981). The present study found that at 24 and 48 h, the mean pain score of the three groups was almost the same and did not differ significantly.

### 3.1 Limitations

This study had some limitations. The subjective nature of pain might make it difficult to compare groups accurately. Furthermore, the COVID‐19 pandemic and strict eligibility criteria resulted in a relatively small sample size. Additionally, secondary outcomes such as patient compliance and side effects were not predefined.

### 3.2 Recommendations

Future studies are required on the analgesic efficacy of ANAHEAL and ANAHEAL PLUS for pain control after archwire placement. Besides, their effect on OTM should be investigated in future studies.

## 4 Introduction

Pain is a widely reported side effect of orthodontic tooth movement (OTM), which can adversely affect patient compliance with treatment, deter the patients from commencing or continuing their treatment (Topolski et al. 2018).

Orthodontic pain is defined by patients as a sense of discomfort, hypersensitivity, dull pain, soreness, tension, or pressure in the affected teeth, which is induced by OTM. The prevalence of orthodontic pain reportedly ranges from 72% to 100% (Panda et al. 2015). Orthodontic forces applied to the teeth activate sensory receptors in the periodontal ligament (PDL), triggering a sequence of events that involves nociceptive pain transduction in both the central and peripheral nervous systems, ultimately leading to the patient's perception of pain (Tuncer et al. 2011)Various orthodontic procedures, including separator insertion, banding, wire engagement, elastic placement, rapid maxillary expansion, and bracket debonding, may be linked to pain (Panda et al. 2015; Tuncer et al. 2011). Orthodontic pain generally commences 12 h following the application of orthodontic force, reaches its peak at 24 h, progressively diminishes over 3–7 days, and is entirely resolved after 1 month (Markovic et al. 2015; Wang et al. 2012).

Despite the significance of orthodontic pain for both patients and orthodontists, a consensus on a standardized specific strategy to mitigate pain induced by orthodontic appliances has not been established. Several modalities have been proposed to alleviate orthodontic pain, including pharmaceutical therapy (Gupta et al. 2014; Xiaoting et al. 2010), mechanical approaches (Woodhouse et al. 2015), laser therapy, and behavioral measures (Woodhouse et al. 2015). Analgesics, especially nonsteroidal anti‐inflammatory medicines (NSAIDs), are frequently used for the management of orthodontic pain. However, some concerns exist regarding the deceleration of OTM caused by the administration of NSAIDs (Arias and Marquez‐Orozco 2006; Ashkenazi et al. 2012; Krasny et al. 2013). NSAIDs inhibit prostaglandin synthesis, which could suppress the osteoclasts and the bone remodeling process, adversely affect the rate of OTM (Arias and Marquez‐Orozco 2006). Moreover, NSAIDs interfere with the activity of collagenase and procollagen synthesis and prevent periodontal remodeling (Kyrkanides et al. 2000). Besides, NSAIDs might cause side effects, such as gastrointestinal difficulties, renal function disturbances, decreased platelet function, dyspnea, and hypotension (Piecuch 2012).

Bromelain is a proteolytic enzyme extracted from the fruit and stem of pineapple. It has long been used in traditional medicine in Southeast Asia, India, Kenya, and China because of its anti‐inflammatory, anti‐thrombotic, anti‐fibrinolytic, and anti‐edema properties. Bromelain contains endopeptidases, thiols, phosphatase, glucosidase, peroxidase, glycoproteins, and protease inhibitors (Mameli et al. 2020; Saptarini et al. 2019). Bromelain was shown to be efficiently absorbed by the human body after oral administration and has no significant side effects, even after long‐term use (Moffa et al. 2019). In dentistry, Bromelain has been used for its anti‐inflammatory properties, particularly following third molar extraction, in comparison to other anti‐inflammatory medicines. Researchers Liu et al. wanted to know that Bromelain may help reduce trismus, discomfort, and edema in the face of people getting impacted by third molar surgery. They came to the conclusion that Bromelain helps reduce swelling in the face both immediately following surgery and in the later phases. Although Bromelain reduced postoperative pain 7 days following surgery, it did not significantly alter pain levels in the initial phases. Bromelain did not seem to affect either early or late trismus, according to the study(Liu et al. 2019).

Turmeric, scientifically called *Curcuma longa*, is a medicinal herb extensively employed in the culinary, cosmetic, and pharmaceutical sectors. Curcumin is the principal component of turmeric, accountable for most of its characteristics, including anti‐inflammatory, antioxidant, anti‐mutagenic, and antibacterial activities (Kuwatada et al. 2017). It also serves as a natural analgesic due to its anti‐inflammatory properties, and it enhances wound healing (Kuwatada et al. 2017). The anti‐inflammatory activity of curcumin is attributed to the inhibition of prostaglandin production and prevention of COX‐2 expression (Aggarwal et al. 2003). Researchers Tantry Maulina et al. found that curcumin effectively reduced acute inflammation and pain following surgery to remove impacted third molars and concluded curcumin effectively alleviated the discomfort associated with acute inflammation in patients with impacted third molars (Maulina et al. 2018).

Concerning the increasing popularity of herbal medications, this study aimed to compare the analgesic efficacy of ANAHEAL tablets containing 200 mg Bromelain and ANAHEAL PLUS tablets containing 150 mg Bromelain and 300 mg Turmeric (95% curcumin) to 400 mg Ibuprofen for pain control following the placement of orthodontic separators.

## 5 Conclusion

Bromelain with/without turmeric might be used as an effective alternative to Ibuprofen for pain control following orthodontic separator placement.

## Author Contributions

**Shabnam Ajami** and **Neda Babanouri:** study design, concept and writing article. **Seyyed Hadi Hosseini:** performing the study, data collection and drafting. **Zahra Hashemi:** sample collection and analysis. **Neda Babanouri:** supervising. All authors read and approved the study.

## Ethics Statement

The study was approved by the Ethics Committee of Shiraz University of Medical Sciences, (IR.SUMS.DENTAL.REC.1399.067).

## Conflicts of Interest

The authors declare no conflicts of interest.

## Data Availability

Data are available upon request from the corresponding author.

## References

[cre270124-bib-0001] Abdaljawwad, A. A. M. , Z. H. Al_Taee , and L. I. Sood . 2016. “The Effect of Meloxicam and Mefenamic Acid Premedication on Pain Experience in Orthodontic Patients.” Iraqi Dental Journal 38, no. 3: 147–153.

[cre270124-bib-0002] Abdelrahman, R. S. , K. S. Al‐Nimri , and E. F. Al Maaitah . 2015. “Pain Experience During Initial Alignment With Three Types of Nickel‐Titanium Archwires: A Prospective Clinical Trial.” Angle Orthodontist 85, no. 6: 1021–1026. 26516711 10.2319/071614-498.1 PMC8612050

[cre270124-bib-0003] Aggarwal, B. B. , A. Kumar , and A. C. Bharti . 2003. “Anticancer Potential of Curcumin: Preclinical and Clinical Studies.” Anticancer Research 23, no. 1/A: 363–398. 12680238

[cre270124-bib-0004] Al‐Hanbali, L. M. S. , A. S. Burhan , M. Y. Hajeer , K. Sultan , and F. R. Nawaya . 2024. “The Effectiveness of Interventions in Reducing Pain Related to Orthodontic Separation: A Systematic Review and Meta‐Analysis.” European Journal of Orthodontics 46, no. 1: cjad078. 38168817 10.1093/ejo/cjad078

[cre270124-bib-0005] Arias, O. R. , and M. C. Marquez‐Orozco . 2006. “Aspirin, Acetaminophen, and Ibuprofen: Their Effects on Orthodontic Tooth Movement.” American Journal of Orthodontics and Dentofacial Orthopedics 130, no. 3: 364–370. 10.1016/j.ajodo.2004.12.027. 16979495

[cre270124-bib-0006] Ashkenazi, M. , Y. Berlin‐Broner , and L. Levin . 2012. “Pain Prevention and Management During Orthodontic Treatment as Perceived by Patients.” Orthodontics: The Art and Practice of Dentofacial Enhancement 13, no. 1: 76–81. 22567657

[cre270124-bib-0007] Bergius, M. , U. Berggren , and S. Kiliaridis . 2002. “Experience of Pain During an Orthodontic Procedure.” European Journal of Oral Sciences 110, no. 2: 92–98. 10.1034/j.1600-0722.2002.11193.x. 12013568

[cre270124-bib-0008] Bird, S. E. , K. Williams , and K. Kula . 2007. “Preoperative Acetaminophen vs Ibuprofen for Control of Pain After Orthodontic Separator Placement.” American Journal of Orthodontics and Dentofacial Orthopedics 132, no. 4: 504–510. 10.1016/j.ajodo.2006.11.019. 17920504

[cre270124-bib-0009] Brignardello‐Petersen, R. 2019. “Curcumin Probably Does Not Reduce Pain Importantly After Impacted Mandibular Third‐Molar Surgery Compared With Mefenamic Acid.” Journal of the American Dental Association 150, no. 1: e7. 30143229 10.1016/j.adaj.2018.06.025

[cre270124-bib-0010] Cheng, C. , T. Xie , and J. Wang . 2020. “The Efficacy of Analgesics in Controlling Orthodontic Pain: A Systematic Review and Meta‐Analysis.” BMC Oral Health 20, no. 1: 259. 10.1186/s12903-020-01245-w. 32948150 PMC7501721

[cre270124-bib-0011] Cooper, S. A. 1981. “Comparative Analgesic Efficacies of Aspirin and Acetaminophen.” Archives of Internal Medicine 141, no. 3: 282–285. 7008731 10.1001/archinte.141.3.282

[cre270124-bib-0012] Ertan Erdinc, A. M. 2004. “Perception of Pain During Orthodontic Treatment With Fixed Appliances.” European Journal of Orthodontics 26, no. 1: 79–85. 10.1093/ejo/26.1.79. 14994886

[cre270124-bib-0013] Farzanegan, F. , S. M. Zebarjad , S. Alizadeh , and F. Ahrari . 2012. “Pain Reduction After Initial Archwire Placement in Orthodontic Patients: A Randomized Clinical Trial.” American Journal of Orthodontics and Dentofacial Orthopedics 141, no. 2: 169–173. 10.1016/j.ajodo.2011.06.042. 22284284

[cre270124-bib-0014] Gupta, M. , S. Kandula , S. M. Laxmikanth , S. S. Vyavahare , S. B. H. Reddy , and C. S. Ramachandra . 2014. “Controlling Pain During Orthodontic Fixed Appliance Therapy With Non‐Steroidal Anti‐Inflammatory Drugs (NSAID): A Randomized, Double‐Blinded, Placebo‐Controlled Study.” Journal of Orofacial Orthopedics/Fortschritte der Kieferorthopädie 75, no. 6: 471–476. 10.1007/s00056-014-0243-7. 25355194

[cre270124-bib-0015] Hikisz, P. , and J. Bernasinska‐Slomczewska . 2021. “Beneficial Properties of Bromelain.” Nutrients 13, no. 12: 4313. 34959865 10.3390/nu13124313 PMC8709142

[cre270124-bib-0016] Hosseinzadeh Nik, T. , N. Shahsavari , H. Ghadirian , and S. N. Ostad . 2016. “Acetaminophen Versus Liquefied Ibuprofen for Control of Pain During Separation in Orthodontic Patients: A Randomized Triple Blinded Clinical Trial.” Acta Medica Iranica 54, no. 7: 418–421. 27424011

[cre270124-bib-0017] Jones, M. , and C. Chan . 1992. “The Pain and Discomfort Experienced During Orthodontic Treatment: A Randomized Controlled Clinical Trial of Two Intial Aligning Arch Wires.” American Journal of Orthodontics and Dentofacial Orthopedics 102, no. 4: 373–381. 1456222 10.1016/0889-5406(92)70054-e

[cre270124-bib-0018] Kluemper, G. T. , D. G. Hiser , M. K. Rayens , and M. J. Jay . 2002. “Efficacy of a Wax Containing Benzocaine in the Relief of Oral Mucosal Pain Caused by Orthodontic Appliances.” American Journal of Orthodontics and Dentofacial Orthopedics 122, no. 4: 359–365. 10.1067/mod.2002.126405. 12411880

[cre270124-bib-0019] Krasny, M. , M. Zadurska , G. Cessak , and P. Fiedor . 2013. “Analysis of Effect of Non‐Steroidal Anti‐Inflammatory Drugs on Teeth and Oral Tissues During Orthodontic Treatment. Report Based on Literature Review.” Acta Poloniae Pharmaceutica 70, no. 3: 573–577. 23757949

[cre270124-bib-0020] Kuwatada, J. S. , M. Raja , and P. Sood . 2017. “Turmeric: A Boon to Oral Health.” International Journal of Oral Care & Research 5, no. 4: 338–341.

[cre270124-bib-0021] Kyrkanides, S. , M. K. O'Banion , and J. D. Subtelny . 2000. “Nonsteroidal Anti‐Inflammatory Drugs in Orthodontic Tooth Movement: Metalloproteinase Activity and Collagen Synthesis by Endothelial Cells.” American Journal of Orthodontics and Dentofacial Orthopedics 118, no. 2: 203–209. 10.1067/mod.2000.105872. 10935962

[cre270124-bib-0022] Leelakanok, N. , A. Petchsomrit , T. Janurai , C. Saechan , and N. Sunsandee . 2023. “Efficacy and Safety of Bromelain: A Systematic Review and Meta‐Analysis.” Nutrition and Health 29, no. 3: 479–503. 10.1177/02601060231173732. 37157782

[cre270124-bib-0023] Liu, S. , H. Zhao , Y. Wang , H. Zhao , and C. Ma . 2019. “Oral Bromelain for the Control of Facial Swelling, Trismus, and Pain After Mandibular Third Molar Surgery: A Systematic Review and Meta‐Analysis.” Journal of Oral and Maxillofacial Surgery 77, no. 8: 1566–1574. 10.1016/j.joms.2019.02.044. 30986376

[cre270124-bib-0024] Mameli, A. , V. Natoli , and C. Casu . 2020. “Bromelain: An Overview of Applications in Medicine and Dentistry.” Biointerface Research in Applied Chemistry 11: 8165–8170.

[cre270124-bib-0025] Markovic, E. , J. Fercec , I. Scepan , et al. 2015. “The Correlation Between Pain Perception Among Patients With Six Different Orthodontic Archwires and the Degree of Dental Crowding.” Srpski Arhiv za Celokupno Lekarstvo 143, no. 3–4: 134–140. 10.2298/sarh1504134m. 26012120

[cre270124-bib-0026] Maulina, T. , H. Diana , A. Cahyanto , and A. Amaliya . 2018. “The Efficacy of Curcumin in Managing Acute Inflammation Pain on the Post‐Surgical Removal of Impacted Third Molars Patients: A Randomised Controlled Trial.” Journal of Oral Rehabilitation 45, no. 9: 677–683. 10.1111/joor.12679. 29908031

[cre270124-bib-0027] Meghana, M. S. , J. Deshmukh , M. Devarathanamma , K. Asif , L. Jyothi , and H. Sindhura . 2020. “Comparison of Effect of Curcumin Gel and Noneugenol Periodontal Dressing in Tissue Response, Early Wound Healing, and Pain Assessment Following Periodontal Flap Surgery in Chronic Periodontitis Patients.” Journal of Indian Society of Periodontology 24, no. 1: 54–59. 31983846 10.4103/jisp.jisp_105_19 PMC6961441

[cre270124-bib-0028] Moffa, A. , F. Fraccaroli , S. Carbone , et al. 2019. “Bromelain After Oral or Dental Procedures: An Update.” Journal of Biological Regulators and Homeostatic Agents 33, no. 5: 1629–1634. 31507135

[cre270124-bib-0029] Panda, S. , V. Verma , A. Sachan , and K. Singh . 2015. “Perception of Pain Due to Various Orthodontic Procedures.” Quintessence International (Berlin, Germany: 1985) 46, no. 7: 603–609. 25918756 10.3290/j.qi.a33933

[cre270124-bib-0030] Piecuch, J. F. 2012. “What Strategies Are Helpful in the Operative Management of Third Molars?” Journal of Oral and Maxillofacial Surgery 70, no. 9 S1: S25–S32. 10.1016/j.joms.2012.04.027. 22916697

[cre270124-bib-0031] Polat, O. , A. I. Karaman , and E. Durmus . 2005. “Effects of Preoperative Ibuprofen and Naproxen Sodium on Orthodontic Pain.” Angle Orthodontist 75, no. 5: 791–796. 10.1043/0003-3219(2005)75[791:Eopian]2.0.Co;2. 16279825

[cre270124-bib-0032] Saptarini, N. , D. Rahayu , and I. Herawati . 2019. “Antioxidant Activity of Crude Bromelain of Pineapple (*Ananas comosus* (L.) Merr) Crown From Subang District, Indonesia.” Journal of Pharmacy and BioAllied Sciences 11, no. S4: 551. 10.4103/jpbs.JPBS_200_19. PMC7020843 32148362

[cre270124-bib-0033] Sharma, S. , B. Singh , A. K. Shahi , S. Chandra , B. D. Kumar , and R. N. Singh . 2022. “Separation Effect and Perception of Pain and Discomfort From Kesling and Elastomeric Orthodontic Separators: An In Vivo Study.” Journal of Contemporary Dental Practice 23, no. 5: 508–512. 35986458

[cre270124-bib-0034] Sterniczuk, B. , P. E. Rossouw , D. Michelogiannakis , and F. Javed . 2022. “Effectiveness of Curcumin in Reducing Self‐Rated Pain‐Levels in the Orofacial Region: A Systematic Review of Randomized‐Controlled Trials.” International Journal of Environmental Research and Public Health 19, no. 11: 6443. 35682028 10.3390/ijerph19116443 PMC9180889

[cre270124-bib-0035] Sudhakar, V. , T. Vinodhini , A. Mohan , B. Srinivasan , and B. Rajkumar . 2014. “The Efficacy of Different Pre‐ and Post‐Operative Analgesics in the Management of Pain After Orthodontic Separator Placement: A Randomized Clinical Trial.” Journal of Pharmacy and BioAllied Sciences 6, no. S1: 80. 10.4103/0975-7406.137393. PMC4157287 25210391

[cre270124-bib-0036] Thejasri, K. , G. S. Singaraju , A. Marya , J. S. Y. Priyanka , S. Shaik , and P. Mandava . 2023. “Separation Effect, Pain Perception During Functional Activity and Gingival Inflammation of Elastomeric and Kansal Separators—A Split Mouth Study.” Clinical Oral Investigations 27, no. 10: 6015–6026. 37648798 10.1007/s00784-023-05215-8

[cre270124-bib-0037] Topolski, F. , A. Moro , G. M. Correr , and S. C. Schimim . 2018. “Optimal Management of Orthodontic Pain.” Journal of Pain Research 11: 589–598. 10.2147/JPR.S127945. 29588616 PMC5859910

[cre270124-bib-0038] Tripathi, T. , N. Singh , P. Rai , and N. Khanna . 2019. “Separation and Pain Perception of Elastomeric, Kesling and Kansal Separators.” Dental Press Journal of Orthodontics 24: 42–48. 31116286 10.1590/2177-6709.24.2.042-048.oar PMC6526766

[cre270124-bib-0039] Tuncer, Z. , F. S. Ozsoy , and O. Polat‐Ozsoy . 2011. “Self‐Reported Pain Associated With the Use of Intermaxillary Elastics Compared to Pain Experienced After Initial Archwire Placement.” Angle Orthodontist 81, no. 5: 807–811. 10.2319/092110-550.1. 21446869 PMC8916199

[cre270124-bib-0040] Viganò, L. , C. Casu , and F. Argenta . 2017. “Evaluation of the Efficacy of Bromelain in Oral and Implant Surgery Patients.” Italian Journal of Dental Medicine 2, no. 3: 83–89.

[cre270124-bib-0041] Wang, J. , F. Jian , J. Chen , et al. 2012. “Cognitive Behavioral Therapy for Orthodontic Pain Control: A Randomized Trial.” Journal of Dental Research 91, no. 6: 580–585. 10.1177/0022034512444446. 22492277

[cre270124-bib-0042] Woodhouse, N. R. , A. T. DiBiase , S. N. Papageorgiou , et al. 2015. “Supplemental Vibrational Force Does Not Reduce Pain Experience During Initial Alignment With Fixed Orthodontic Appliances: A Multicenter Randomized Clinical Trial.” Scientific Reports 5: 17224. 10.1038/srep17224. 26610843 PMC4661602

[cre270124-bib-0043] Xiaoting, L. , T. Yin , and C. Yangxi . 2010. “Interventions for Pain During Fixed Orthodontic Appliance Therapy. A Systematic Review.” Angle Orthodontist 80, no. 5: 925–932. 10.2319/010410-10.1. 20578865 PMC8939023

[cre270124-bib-0044] Young, A. N. , R. W. Taylor , S. E. Taylor , S. A. Linnebur , and P. H. Buschang . 2006. “Evaluation of Preemptive Valdecoxib Therapy on Initial Archwire Placement Discomfort in Adults.” Angle Orthodontist 76, no. 2: 251–259. 16539549 10.1043/0003-3219(2006)076[0251:EOPVTO]2.0.CO;2

[cre270124-bib-0045] Zarif Najafi, H. , M. Oshagh , P. Salehi , N. Babanouri , and S. Torkan . 2015. “Comparison of the Effects of Preemptive Acetaminophen, Ibuprofen, and Meloxicam on Pain After Separator Placement: A Randomized Clinical Trial.” Progress in Orthodontics 16: 34. 10.1186/s40510-015-0104-y. 26467790 PMC4605934

